# Supplementary material for: Erythroid differentiation regulator-1 induced by microbiota in early life drives intestinal stem cell proliferation and regeneration
Source: Nat Commun. 2020 Jan 24;11:513. doi: 10.1038/s41467-019-14258-z (PMC6981263; doi:10.1038/s41467-019-14258-z)
Supplement: Supplementary file 1 — Supplementary Information [file 41467_2019_14258_MOESM1_ESM.pdf]

Supplementary Information

Erythroid differentiation regulator-1 induced by microbiota in early-life drives intestinal stem cell proliferation and regeneration

Abo et al.

This PDF file includes supplementary figure 1 to 16

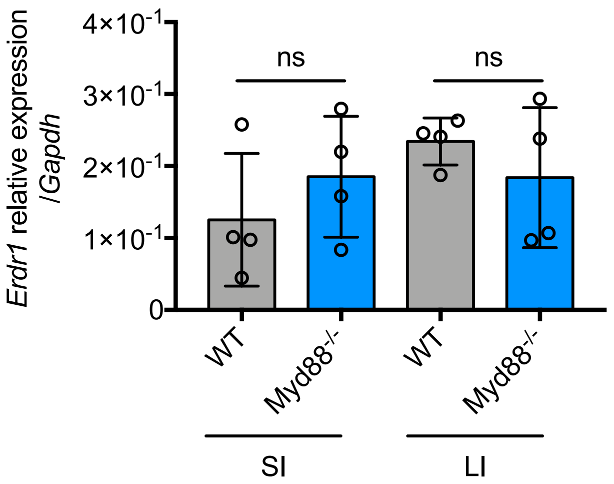

Supplementary Figure 1 | Erdr1 expression is independent of Myd88 in the small and large intestine.

Expression of Erdr1 in total intestinal tissue from the indicated mice. n=4; Data are presented as mean ± SD; ns, no significance by unpaired, two-tailed *t*-tests.

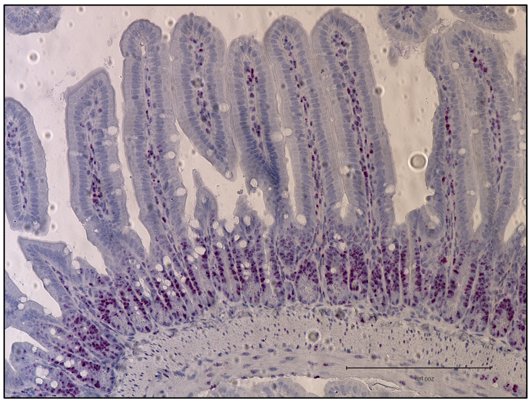

ISH: Erdr1 200 μm

Supplementary Figure 2 | RNAscope for Erdr1 in mouse small intestine.

RNAscope staining for *Erdr1* within small intestine from SPF mice. Purple dots indicate *Erdr1* RNA.

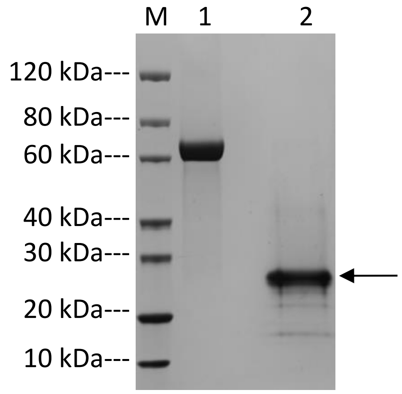

Supplementary Figure 3 | SDS-PAGE gel of recombinant Erdr1 staind by coomassie blue.

Lane M;Protein marker, Lane 1;BSA, Lane;2 Erdr1.

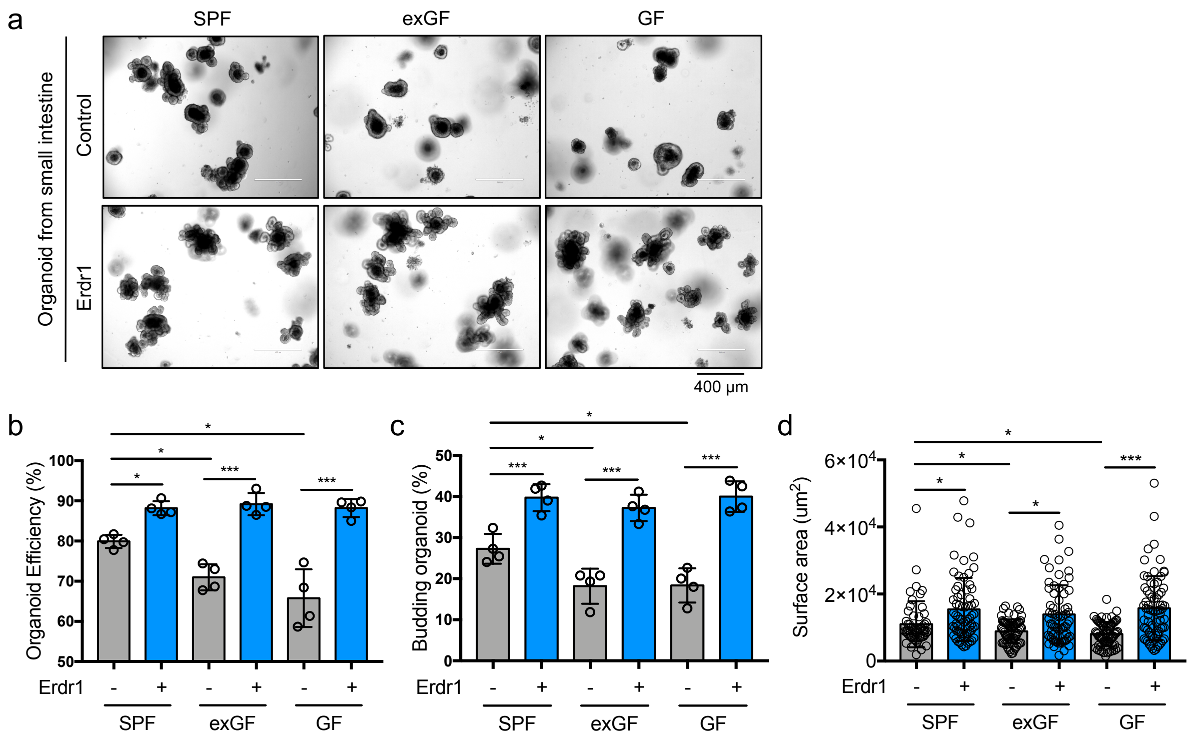

Supplementary Figure 4 | Erdr1 increases growth of small intestine organoids.

a, Representative images of small intestine (SI) organoids derived from SPF, exGF and GF mice and cultured +/- Erdr1 (1 μg) for 6 days. **b,** Organoid efficiency of SI organoids cultured +/- Erdr1. n=4; representative of three experiments. **c,** Frequency of budding organoids cultured +/- Erdr1 for 6 days. n=4; representative of three experiments. **d,** Surface area of SI organoids cultured +/- Erdr1. n=55 (SPF), n=67 (exGF), n=63 (GF); representative of three experiments. All data are presented as mean ± SD; *P <0.05, **P <0.01, ***P <0.001 by unpaired, two-tailed t-tests.

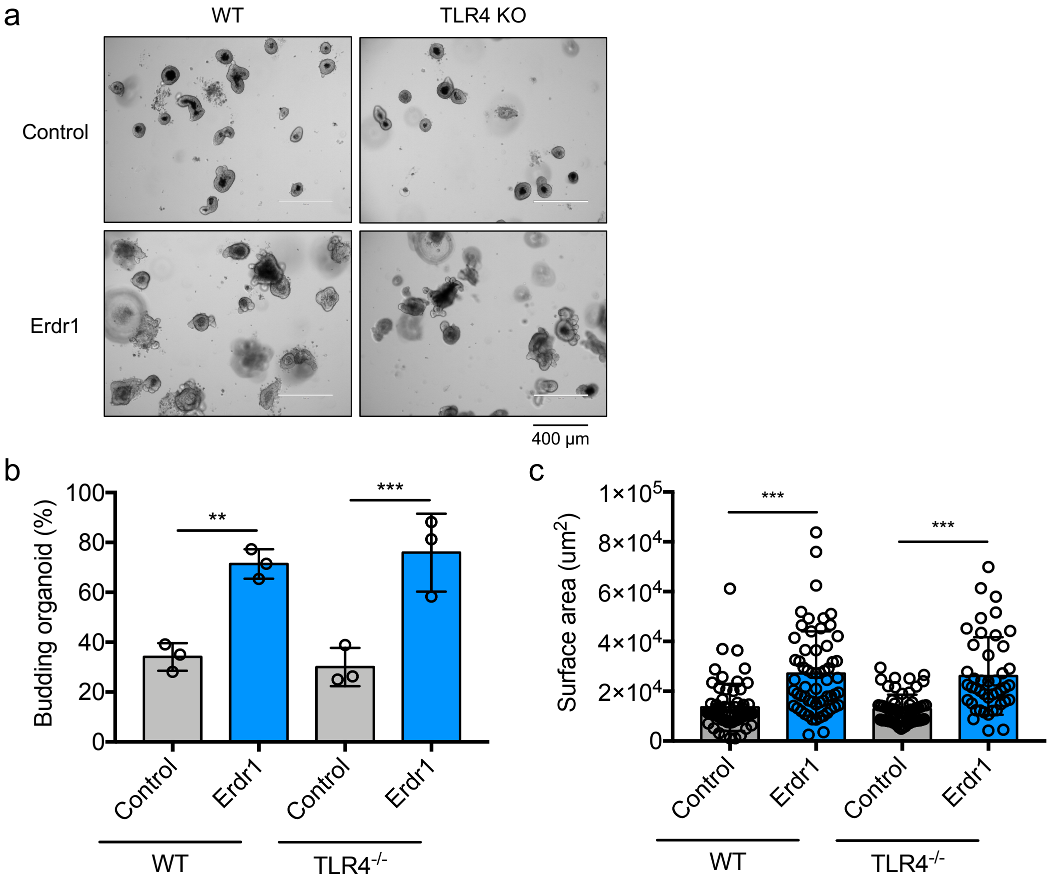

Supplementary Figure 5 | TLR4 signaling does not influence organoid growth.

a, Representative images of LI organoids derived from WT and TLR4 KO mice and cultured with or without Erdr1 for 6 days. **b,** Frequency of budding organoids with or without Erdr1. n=3; representative of two experiments. **c,** Surface area of LI organoids cultured with or without Erdr1. n=67 (WT/control), n=62 (WT/Erdr1), n=58 ($TLR4^{-/-}$/control), n=63 ($TLR4^{-/-}$/Erdr1); representative of two experiments. All data are presented as mean ± SD; $^{**}P$ <0.01, $^{***}P$ <0.001 by one-way ANOVA with Tukey's multiple comparison test.

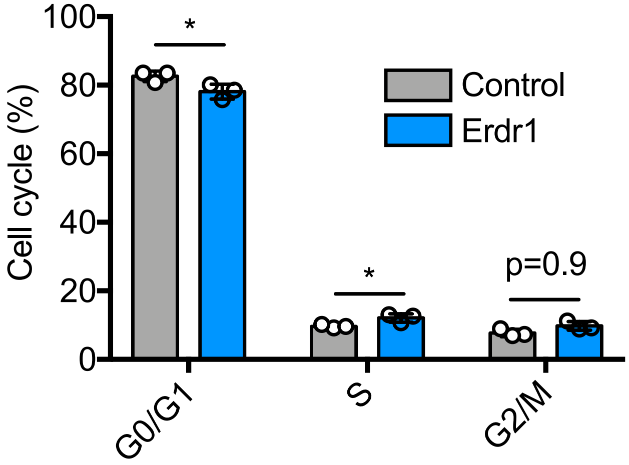

Supplementary Figure 6 | Cell cycle analysis of small intestine organoids cultured +/- Erdr1.

Cell cycle analysis of small intestine (SI) organoids cultured +/- Erdr1 (1 µg/mL). n=3; representative of two experiments. All data are presented as mean ± SD; **P* <0.05, two-tailed *t*-tests.

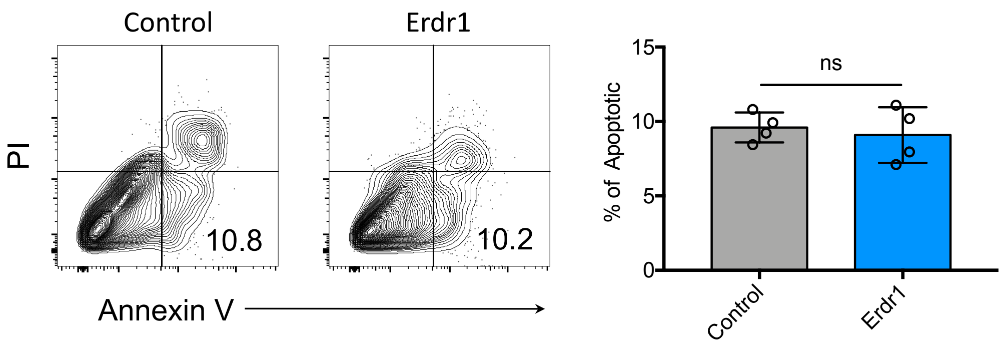

Supplementary Figure 7 | Erdr1 does not influence apoptosis in organoid cultures.

Frequency of pre-apoptotic cells in organoids derived from large intestine cultures with or without Erdr1. n=4; representative of two experiments. Data are presented as mean ± SD; ns, no significant by unpaired, two- tailed *t*-tests.

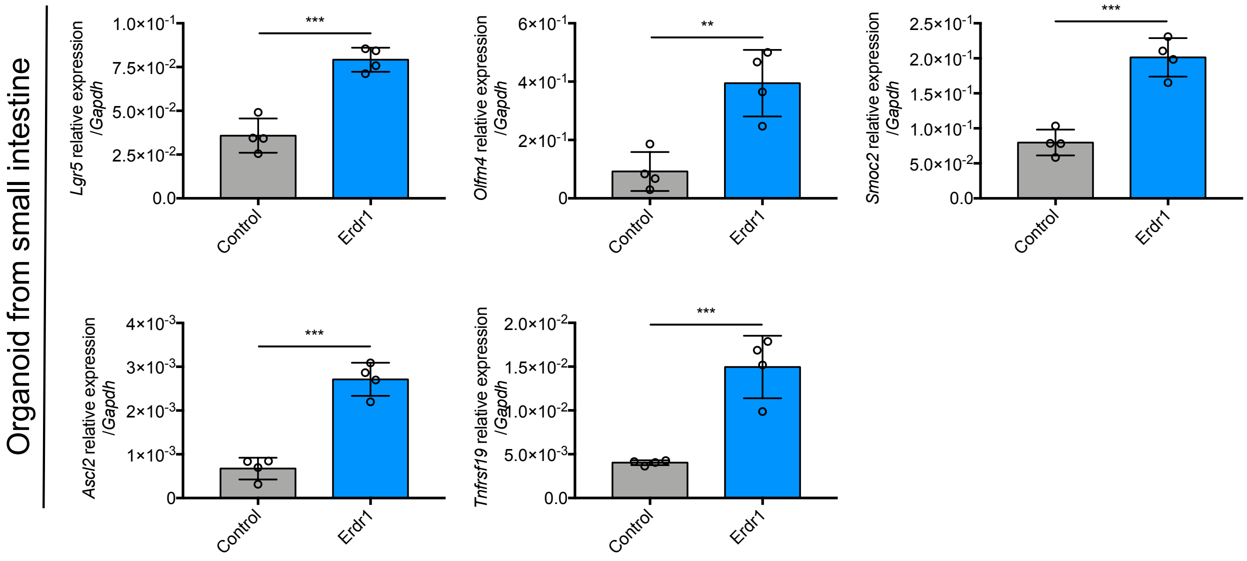

Supplementary Figure 8 | Erdr1 induces ISC signature gene expression.

a, Expression of stem cell signature genes (*Lgr5*, *Olmf4*, *Smoc2*, *Ascl2*, *Tnfrsf19*) in SI organoids cultured +/- Erdr1 for 6 days. n=4; representative of three experiments. All data are presented as mean ± SD; ***P* <0.01, ****P* <0.001 by unpaired, two-tailed *t*-tests.

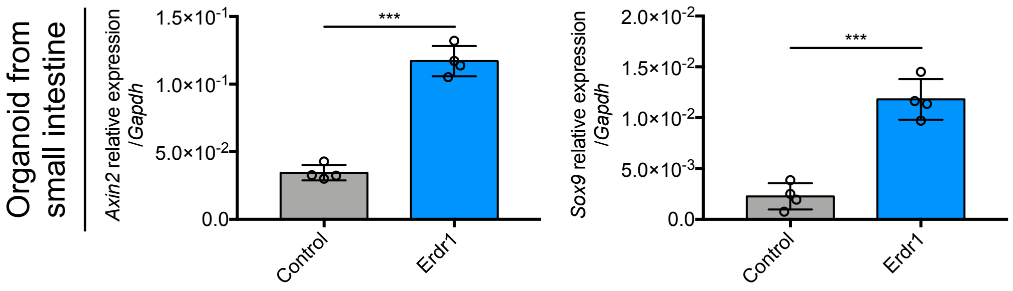

Supplementary Figure 9 | Erdr1 enhances Wnt signaling in small intestine organoids.

Expression of Wnt signaling pathway target genes (*Axin2* and *Sox9*) in SI organoid cultures +/- Erdr1 (1 μg/mL). n=4; representative of three experiments. All data are presented as mean ± SD; ****P* <0.001 by unpaired, two-tailed *t*-tests.

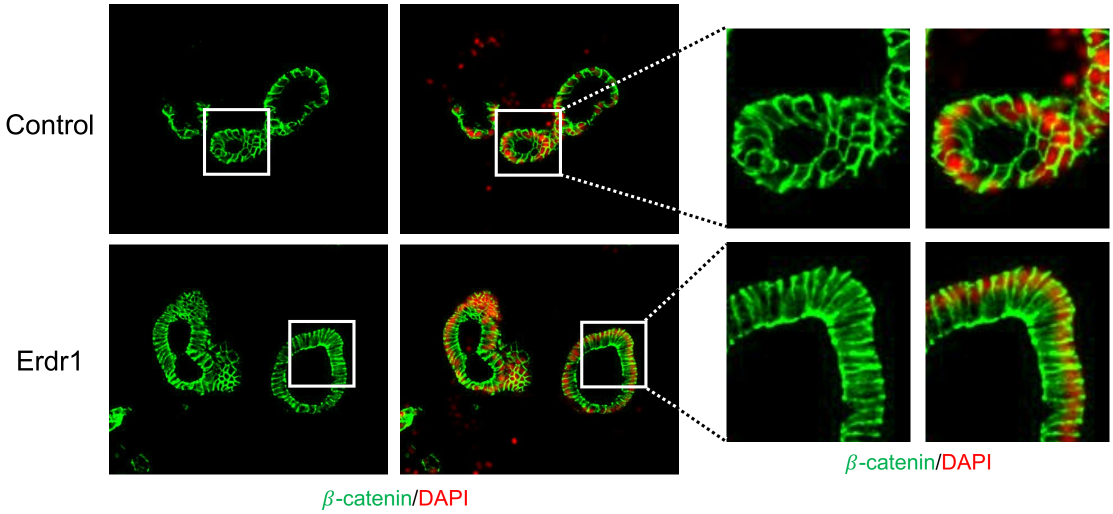

Supplementary Figure 10 | Erdr1 induces nuclear translocation of β-catenin.

Immunofluorescent staining of β-catenin in LI organoid cultures with or without Erdr1 (green, β-catenin; blue, DAPI). Representative of two experiments.

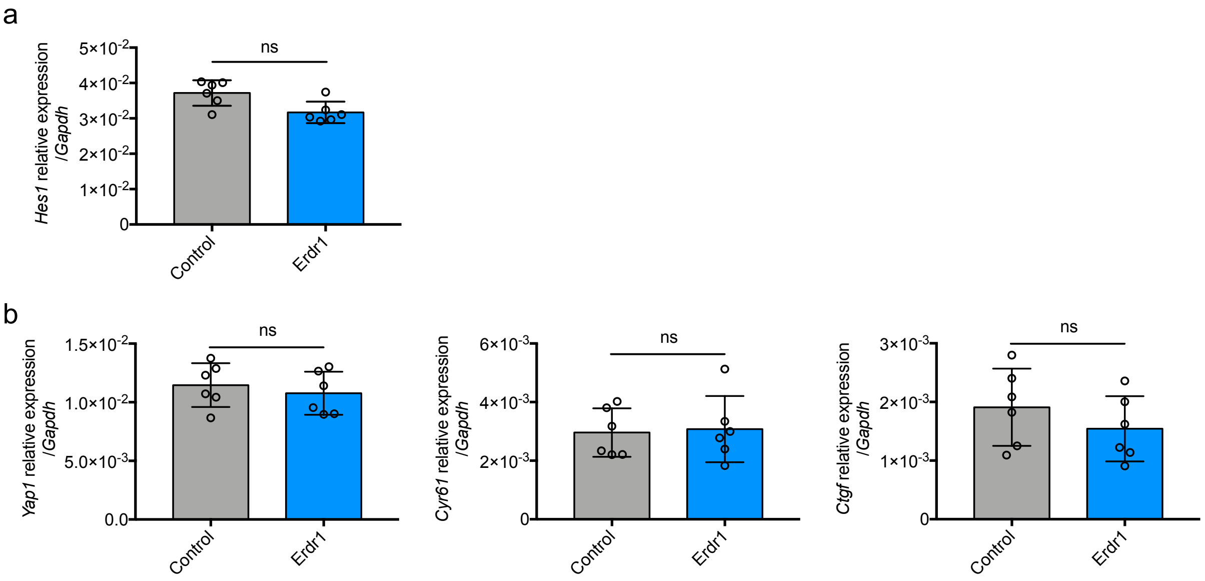

Supplementary Figure 11 | Erdr1 increases organoid growth without inducing Notch or Yap/Taz mRNA expression.

a, Expression of *Hes1* in organoids derived from the large intestine of SPF mice. n=6; two independent experiments. **b,** Expression of *Yap1* and its target genes (*Cyr61, Ctgf*) in organoids derived from the large intestine of SPF mice. n=6; representative of two experiments. Data are presented as mean ± SD; ns, no significant difference (p>0.5) by unpaired, two- tailed t-tests.

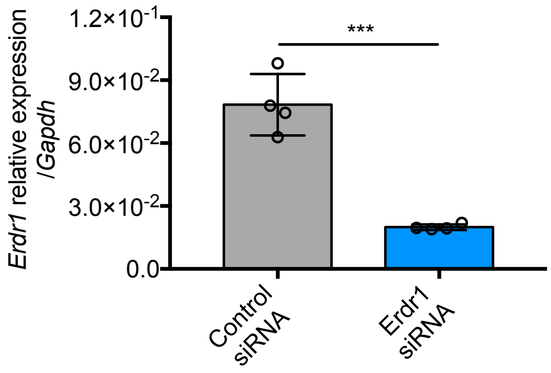

Supplementary Figure 12 | siRNA knockdown of Erdr1.

a, *Erdr1* expression after treatment of Mode-K cells with control siRNA or Erdr1 siRNA for 24 hours. n=4; representative of two experiments. All data are presented as mean ± SD; ****P* <0.001 by unpaired, two-tailed *t*-tests.

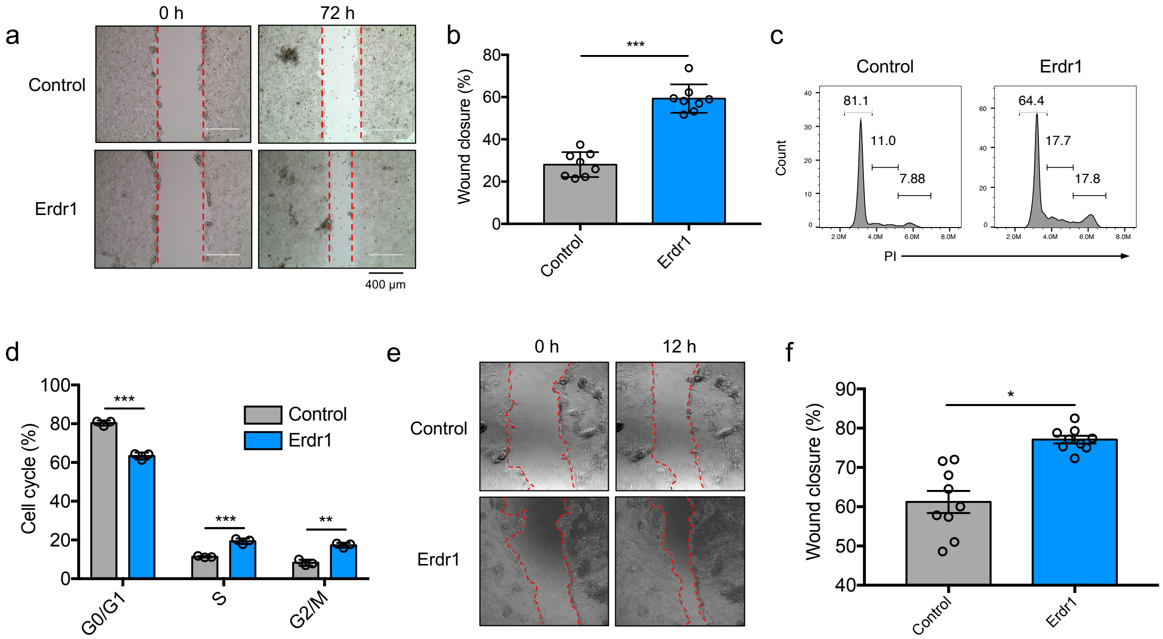

Supplementary Figure 13 | Erdr1 accelerates wound closure in human intestinal epithelial cell.

a-b, Wound closure of HT-29 cells cultured +/- Erdr1 (1 μg/mL) for 72 hours (**a,** picture; **b,** % of closure). n=8; representative of three experiments. **c-d,** Cell cycle analysis of HT-29 cells cultured +/- Erdr1. n=3; representative of three experiments. **e-f,** Wound closure of human 2D organoid monolayers +/- Erdr1 (1 μg/mL) for 12 hours post scratching (**e,** picture; **f,** % of closure). n=9; Data are cumulative of three experiments. All data are presented as mean ± SD; **P* <0.05, ***P* <0.01, ****P* <0.001 by unpaired, two-tailed *t*-tests.

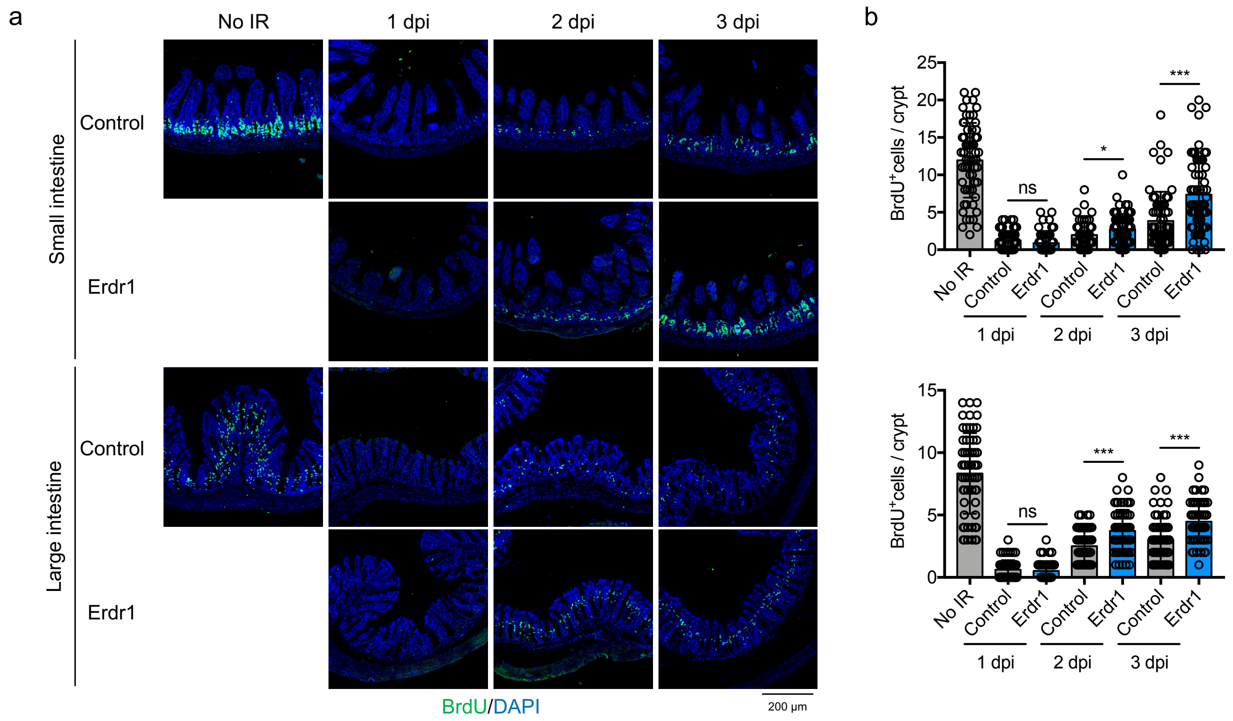

Supplementary Figure 14 | Erdr1 promotes regeneration following radiation-induced injury.

a, Representative images of BrdU staining in ileum and colon sections (green, BrdU; blue, DAPI). Mice were treated with 10 Gy of radiation, and euthanized at the indicated time points. **b,** Quantification of BrdU positive cells per crypt from the SI (top) and LI (bottom). SI, n=60; LI, n=60; All data are presented as mean ± SD; **P* <0.01, ***P* <0.01, ****P* <0.001 by unpaired, two-tailed *t*-tests.

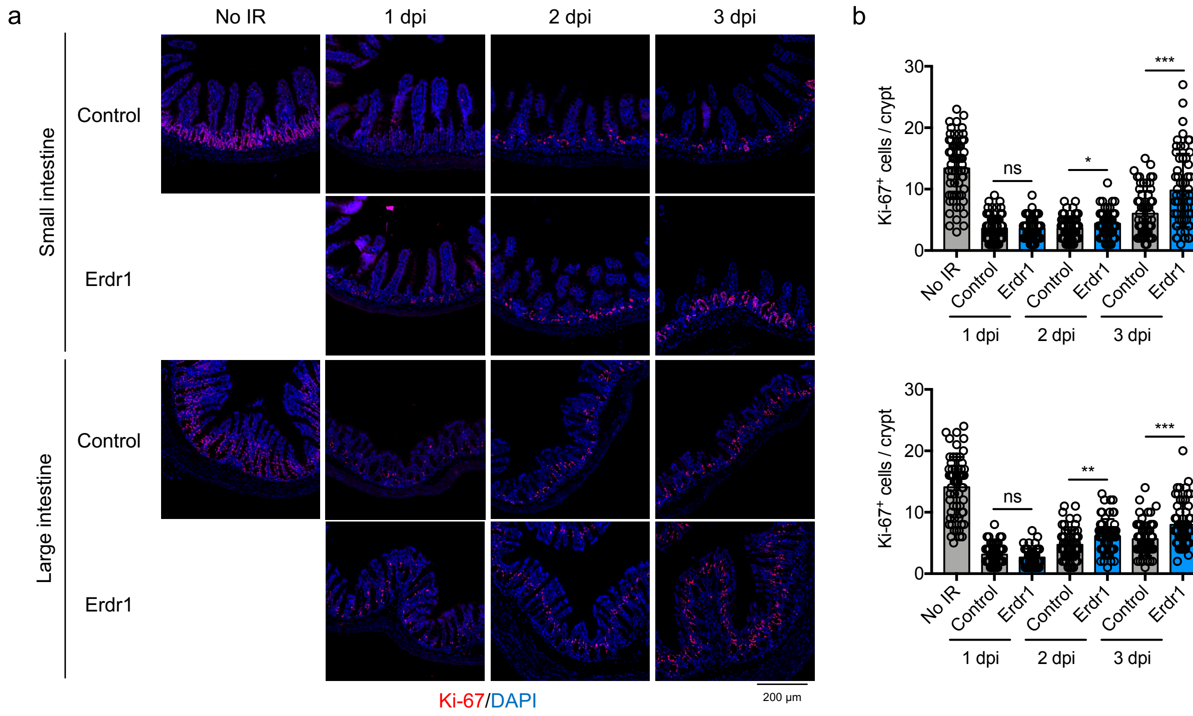

Supplementary Figure 15 | Erdr1 promotes the regeneration following radiation-induced injury.

a, Representative images of Ki-67 staining in ileum and colon sections (pink, Ki-67; blue, DAPI). Mice were treated with 10 Gy of radiation, and euthanized at the indicated time points. **b,** Quantification of Ki-67 positive cells per crypt from the SI (top) and LI (bottom). SI, n=60; LI, n=60; All data are presented as mean ± SD; **P* <0.01, ***P* <0.01, ****P* <0.001 by unpaired, two-tailed *t*-tests.

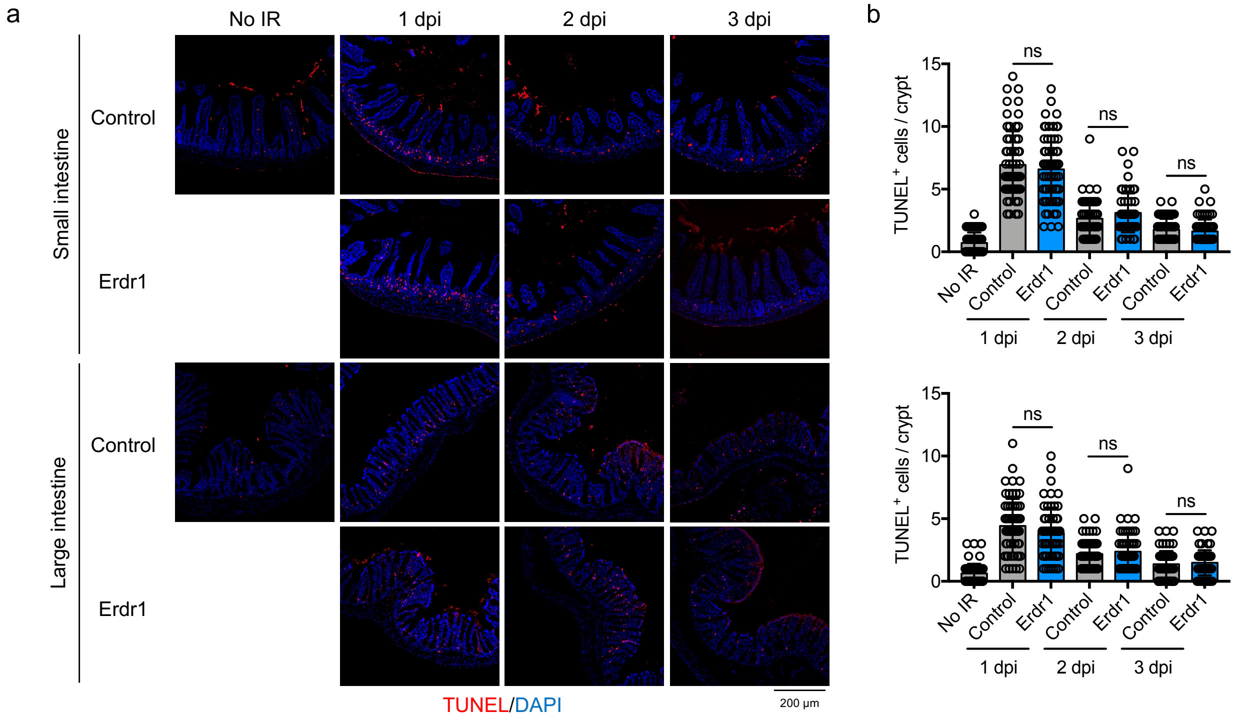

Supplementary Figure 16 | Erdr1 does not influence apoptosis following radiation-induced injury.

a, Representative images of TUNEL staining in ileum and colon sections (pink, TUNEL; blue, DAPI). Mice were treated with 10 Gy of radiation, and euthanized at the indicated time points. **b,** Quantification of TUNEL positive cells per crypt from the SI (top) and LI (bottom). SI, n=60; LI, n=60; All data are presented as mean ± SD; **P* <0.01, ***P* <0.01, ****P* <0.001 by unpaired, two-tailed *t*-tests.
