# Supplementary material for: Erythroid differentiation regulator-1 induced by microbiota in early life drives intestinal stem cell proliferation and regeneration
Source: Nat Commun. 2020 Jan 24;11:513. doi: 10.1038/s41467-019-14258-z (PMC6981263; doi:10.1038/s41467-019-14258-z)
Supplement: Supplementary file 3 — Reporting Summary [file 41467_2019_14258_MOESM3_ESM.pdf]

nature research

Corresponding author(s): Tim Denning
Last updated by author(s): 2019/10/26

Reporting Summary

Nature Research wishes to improve the reproducibility of the work that we publish. This form provides structure for consistency and transparency in reporting. For further information on Nature Research policies, seeAuthors & Referees and theEditorial Policy Checklist .

Statistics

For all statistical analyses, confirm that the following items are present in the figure legend, table legend, main text, or Methods section.

n/a	Confirmed	
☐	☒	The exact sample size (*n*) for each experimental group/condition, given as a discrete number and unit of measurement
☐	☒	A statement on whether measurements were taken from distinct samples or whether the same sample was measured repeatedly
☐	☒	The statistical test(s) used AND whether they are one- or two-sided<br>*Only common tests should be described solely by name; describe more complex techniques in the Methods section.*
☒	☐	A description of all covariates tested
☒	☐	A description of any assumptions or corrections, such as tests of normality and adjustment for multiple comparisons
☐	☒	A full description of the statistical parameters including central tendency (e.g. means) or other basic estimates (e.g. regression coefficient) AND variation (e.g. standard deviation) or associated estimates of uncertainty (e.g. confidence intervals)
☐	☒	For null hypothesis testing, the test statistic (e.g. *F*, *t*, *r*) with confidence intervals, effect sizes, degrees of freedom and *P* value noted<br>*Give P values as exact values whenever suitable.*
☒	☐	For Bayesian analysis, information on the choice of priors and Markov chain Monte Carlo settings
☒	☐	For hierarchical and complex designs, identification of the appropriate level for tests and full reporting of outcomes
☒	☐	Estimates of effect sizes (e.g. Cohen's *d*, Pearson's *r*), indicating how they were calculated

Our web collection on statistics for biologists contains articles on many of the points above.

Software and code

Policy information about availability of computer code

Data collection	Not applicable.
Data analysis	Statistic analysis were performed by GraphPad Prism software. Flowjo was used to analyze flow cytometry data. Organoid size was analyzed by image J software described in methods section.

For manuscripts utilizing custom algorithms or software that are central to the research but not yet described in published literature, software must be made available to editors/reviewers. We strongly encourage code deposition in a community repository (e.g. GitHub). See the Nature Research guidelines for submitting code & software for further information.

Data

Policy information about availability of data

All manuscripts must include a data availability statement. This statement should provide the following information, where applicable:

- Accession codes, unique identifiers, or web links for publicly available datasets
- A list of figures that have associated raw data
- A description of any restrictions on data availability

Source data of this study was provided with the paper for supplementary data.
Analyzed RNAseq data was provided in supplementary data.

Field-specific reporting

Please select the one below that is the best fit for your research. If you are not sure, read the appropriate sections before making your selection.

Life sciences study design

All studies must disclose on these points even when the disclosure is negative.

Sample size	Detail of sample size of all experiments are provided in figure legends.
Data exclusions	No data was excluded from the analysis.
Replication	All experiments were repeated at least twice.
Randomization	All mice were randomized before experiments.
Blinding	Blinding was not performed in this study

Reporting for specific materials, systems and methods

We require information from authors about some types of materials, experimental systems and methods used in many studies. Here, indicate whether each material, system or method listed is relevant to your study. If you are not sure if a list item applies to your research, read the appropriate section before selecting a response.

Materials & experimental systems

n/a	Involved in the study	
☐	☒	Antibodies
☐	☒	Eukaryotic cell lines
☒	☐	Palaeontology
☐	☒	Animals and other organisms
☒	☐	Human research participants
☒	☐	Clinical data

Methods

n/a	Involved in the study	
☒	☐	ChIP-seq
☐	☒	Flow cytometry
☒	☐	MRI-based neuroimaging

Antibodies

Antibodies used	anti-Ki-67-PE (#12-5698-82, invitrogen, 1:200) and anti-EpCAM-APC (#17-5791-82, invitrogen, 1:200) were used for flow cytometry analysis in 1:200 dilution. anti-acetyl-histone H3 (#06-599, EMD Millipore) was used for ChIP assay. anti-GFP (#GFP-1020, Aves, 1:1000), anti-Ki-67 (#ab15580, abcam, 1:500), amti-BrdU (#ab6326, abcam, 1:250), anti-beta catenin (#8814, Cell signaling, 1:500), Alexa fluor 488 conjugated anti-rabbit IgG (#A21206, Invitrogen, 1:1000), Alexa fluor 647 conjugated anti-rat IgG (#A21247, Invitrogen, 1:1000) and Alexa fluor 647 conjugated anti-chiken IgY (#A21449, Invitrogen, 1:1000) were used for immunofluorescence staining.
Validation	All antibody are commercially available and were validated by provider.

Eukaryotic cell lines

Policy information about cell lines

Cell line source(s)	HT-29 cell was provided by Dr. Merlin. SKCO-15 cell was provided by Dr. Nusrat.
Authentication	No authentification was performed.
Mycoplasma contamination	All all cells were negative for mycoplasma contamination.
Commonly misidentified lines (See ICLAC register)	No commonly misidentified cell line were used.

Animals and other organisms

Policy information about studies involving animals; ARRIVE guidelines recommended for reporting animal research

Laboratory animals	C57BL/6, Lgr5-EGFP-IRES-creERT2 and Myd88-/- mice were purchased from the Jackson Laboratory. TLR4-/- mice was provided by Dr. Gewirtz.
Wild animals	Not applicable.
Field-collected samples	Not applicable.

Ethics oversight | All animal experiments were approved by the Institutional Animal Care and Use Committee of Georgia State University.

Note that full information on the approval of the study protocol must also be provided in the manuscript.

Flow Cytometry

Plots

Confirm that:

- [ ] The axis labels state the marker and fluorochrome used (e.g. CD4-FITC).
- [x] The axis scales are clearly visible. Include numbers along axes only for bottom left plot of group (a 'group' is an analysis of identical markers).
- [x] All plots are contour plots with outliers or pseudocolor plots.
- [x] A numerical value for number of cells or percentage (with statistics) is provided.

Methodology

Sample preparation	Samples were prepared as described in methods section.
Instrument	BECKMAN COULTER, CytoFLEX Flow Cytometer
Software	CytoExpert for data collection. Flowjo software version 10 for data analysis.
Cell population abundance	>10,000 cells were analyzed. No sorting experinent in this study.
Gating strategy	All gating strategy started by FSC and SSC area, and live cells.

- [ ] Tick this box to confirm that a figure exemplifying the gating strategy is provided in the Supplementary Information.
