# Supplementary material for: Erythroid differentiation regulator-1 induced by microbiota in early life drives intestinal stem cell proliferation and regeneration
Source: Nat Commun. 2020 Jan 24;11:513. doi: 10.1038/s41467-019-14258-z (PMC6981263; doi:10.1038/s41467-019-14258-z)
Supplement: Supplementary file 4 — Description of Additional Supplementary Files [file 41467_2019_14258_MOESM4_ESM.pdf]

Description of Additional Supplementary Files

File name : Supplementary Data 1
Description : Analyzed data of RNAseq related to Figure 1b.

File name : Supplementary Movie 1
Description : The movie of wound healing assay related to Figure 6a.
